# Time–Temperature–Plasticization Superposition Principle: Predicting Creep of a Plasticized Epoxy

**DOI:** 10.3390/polym11111848

**Published:** 2019-11-09

**Authors:** Andrey E. Krauklis, Anton G. Akulichev, Abedin I. Gagani, Andreas T. Echtermeyer

**Affiliations:** 1 Department of Mechanical and Industrial Engineering, Norwegian University of Science and Technology (NTNU), 7491 Trondheim, Norway; andykrauklis@gmail.com (A.E.K.); anton.akulichev@gmail.com (A.G.A.); abedin.gagani@tuhh.de (A.I.G.); 2 SINTEF Industry, Materials and Nanotechnology, 0314 Oslo, Norway; 3 Strukturplast AS, 6823 Sandane, Norway; 4 Institute of Polymer Composites, Hamburg University of Technology (TUHH), 21073 Hamburg, Germany

**Keywords:** epoxy, creep, water, plasticization, viscoelastic, master curve, time–temperature superposition, time–temperature–plasticization superposition, accelerated testing, methodology

## Abstract

Long-term creep properties and the effect of water are important for fiber reinforced polymer (FRP) composite materials used in offshore applications. Epoxies are often used as a matrix material in such composites. A typical design lifetime of offshore FRP structures is 25 or more years in direct contact with water leading to some deterioration of the material properties. Knowing and predicting the extent of the material property deterioration in water is of great interest for designers and users of the offshore FRP structures. It has been established that the time–temperature superposition principle (TTSP) is a useful tool for estimating changes in properties of polymer materials at long times or extreme temperatures. In this work, a time–temperature–plasticization superposition principle (TTPSP) is described and used for predicting the long-term creep behavior of an epoxy compound. The studied epoxy does not degrade chemically via hydrolysis or chain scission but is negatively affected by plasticization with water. The methodology enables prediction of the long-term viscoelastic behavior of amorphous polymers at temperatures below the glass transition (*T*_g_) using short-term creep experimental data. The results also indicate that it is possible to estimate the creep behavior of the plasticized polymer based on the short-term creep data of the respective dry material and the difference between *T*_g_ values of dry polymer and plasticized polymer. The methodology is useful for accelerated testing and for predicting the time-dependent mechanical properties of a plasticized polymer below the glass transition temperature.

## 1. Introduction

Epoxy polymers are widely used as a constituent matrix material in fiber-reinforced polymer (FRP) composites due to a relatively high specific strength and modulus, low shrinkage on curing, low volatility, and relatively high chemical resistance [1,2]. Bisphenol A diglycidyl ether (DGEBA)-based epoxies, such as used in this work, constitute more than 75% of the epoxide market [3]. FRPs are widely used in structural applications in marine and offshore industries, where a typical design lifetime of composite structures is 25 or more years. During this prolonged service period, the structures are in direct contact with water leading to deterioration of the material properties [4,5,6,7]. Knowing and predicting the extent of the material property deterioration in water is of great interest for designers and users of the offshore FRP structures.

Interaction with water may induce reversible effects (plasticization/hygrothermal) and/or irreversible changes (hygrothermal aging) in epoxy polymers [7,8,9]. While plasticization is usually reversible, irreversible aging processes can occur due to hydrolysis, oxidation, or leaching [1,7]. It has been shown elsewhere that for the studied material, no chain scission is present, while changes due to thermal oxidation and leaching did not have any significant effects on the mechanical properties [6,7,10]. This means saturating the epoxy with water has the effect of plasticization. Plasticization was fully reversible and mechanical properties were fully regained upon redrying the material [6]. When no chain scission is present, such as for this material, a decrease in *T*_g_ is attributed to plasticization – *T*_g_ decreases as the polymer chains become more flexible [5,6,10].

The mechanical behavior of a polymer shows both time and temperature dependence not only above *T*_g_ but also below. This is called viscoelastic behavior [11]. Temperatures below *T*_g_ are within the usual operating temperature range of thermoset FRPs [11].

Thermosets, such as epoxies, are not in thermodynamic equilibrium below *T*_g_, but gradually move towards the equilibrium state [12], so called physical aging. As the polymer is kept at a constant temperature below *T*_g_, its free volume decreases and the state is approaching thermodynamic equilibrium with time [12,13]. The change in free volume of the polymer affects the mechanical behavior of the polymer, e.g., viscoelastic properties [12].

Nakada et al. [13] have shown that the long-term viscoelastic behavior of dry epoxies at temperatures below *T*_g_ can be predicted accurately from measuring the short-term viscoelastic behavior for up to 3 h (the same time is used in this work) at elevated temperatures based on the time–temperature superposition principle (TTSP).

This paper will demonstrate that TTSP is also applicable for epoxy saturated with water. Even more importantly, the TTSP for dry and wet material can be superimposed allowing the generation of a single master curve-termed time–temperature–plasticization superposition principle (TTPSP).

## 2. Theory

### 2.1. Time–Temperature Superposition

**Figure 1:**
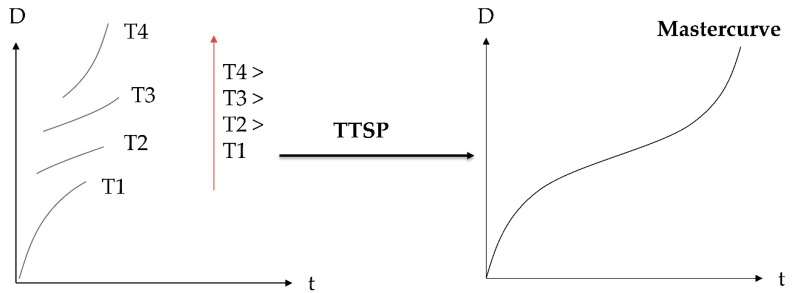
Schematic illustration of the time (t)–temperature (T) superposition principle (TTSP) for the creep compliance (D).

The concept of equivalence of time and temperature has been verified in many polymer systems, primarily single-phase and single-transition amorphous polymers, such as epoxies. For such materials (also called thermo-rheologically simple materials [14,15]) it is possible to establish temperature functions that enable to translate individual isothermal segments of the chosen response function, e.g., creep compliance, along the time scale and compose a master curve, recorded at a reference temperature, *T*_ref_. This approach is called time–temperature superposition principle (TTSP) and it allows to extend the time scale beyond the time limits of convenient testing, giving an accelerated test method. A simple schematic of TTSP is given in Figure 1.

The temperature shifting function, often called temperature shifting factor, can be described by WLF equation [16] in polymers held at temperatures near and above *T*_g_ or Arrhenius types of equation in polymers subjected to temperatures below *T*_g_ (e.g., [15]).

Arrhenius’ equation representing the horizontal shift factor [12] is

(1) $$\log a_{T_{0}}\left(T\right)=\frac{-E_{A}}{2.303R}\left(\frac{1}{T}-\frac{1}{T_{\mathrm{ref}}}\right)$$

where *E*_A_, and *R* are the activation energy, and the gas constant, respectively [12].

### 2.2. Proposed Principle: Time–Temperature–Plasticization Superposition

The specific volume of polymers consists of the occupied volume and the free volume. The occupied volume describes the volume occupied by molecular chains. The free volume is the space that allows movement of these chains [12]. The effect of plasticization acts similarly to that of temperature on the free volume of the polymer [17], thus time–temperature–plasticization superposition principle (TTPSP) can be established. Since *T*_g_ is an indicator of polymer chain mobility and can be related to the free volume [5,17,18], the *T*_g_ values of dry and plasticized polymers can be used to develop a superposition relationship between time, temperature, and concentration of the plasticizing agent [19,20]. Therefore, we hypothesize that the dry-plasticized master curve shift factor should be related to the difference in *T*_g_ of the dry and plasticized polymer according to the Arrhenius equation

(2) $$\log a_{dry-to-plast}=\frac{-E_{A}}{2.303R}\left(\frac{1}{Tg{\;}_{plast}}-\frac{1}{Tg{\;}_{dry}}\right)$$

where $Tg{\;}_{dry}$ and $Tg{\;}_{plast}$ are glass transition temperatures of dry and plasticized material, respectively. $E_{A}$ is the activation energy, the same as used for the TTSP of the dry material (reference state) described in Equation (1).

*T*_g_ is typically defined using standard methods, such as ISO 6721, ASTM E1640, or NS-EN 6032 [21,22,23]. However, in reality, the glass transition temperature is not a well-defined value—the glass transition is gradual within a temperature range and *T*_g_ is located somewhere in this range. Furthermore, *T*_g_ is rate dependent. It depends on the loading rate or heating rate when measuring *T*_g_. Addressing the rate dependence and uncertainty of *T*_g_ properly will be important to keep in mind when applying the shift factor.

**Figure 2:**
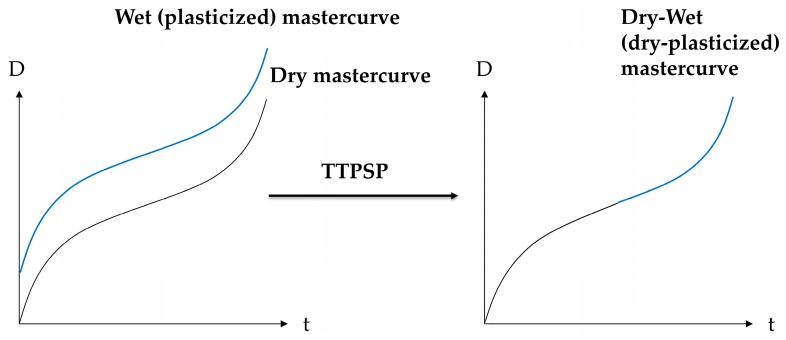
Schematic illustration of the proposed time (t)–temperature (T)–plasticization superposition principle (TTPSP) for the creep compliance D.

The proposed principle of time–temperature–plasticization superposition (TTPSP) is illustrated schematically in Figure 2.

## 3. Materials and Methods

### 3.1. Epoxy Polymer

The amine-cured epoxy polymer was prepared by mixing reagents Epikote Resin RIMR135^TM^ (Hexion, Columbus, OH, USA) and Epikure Curing Agent RIMH137^TM^ (Hexion, Columbus, OH, USA) stoichiometrically (ratio of 100:30 by weight). The mixture was then degassed in a vacuum chamber for 0.5 h in order to remove air bubbles. Degassed resin was poured into an open mold, which had rectangular grooves. The samples were cured at room temperature for 24 h and post-cured in an air oven (Lehmkuhls Verksteder, Oslo, Norway) at 80 °C for 16 h. Full cure was achieved [7]. Samples were rectangular in shape and had dimensions of 40 × 7 × 2 mm. The sample geometry was chosen in accordance with standard practice for glass transition temperature determination [21]. The dimensions were achieved within 5% tolerance.

### 3.2. Conditioning in Water

Water uptake experiments were conducted using a batch system. A PID-controlled heated bath with distilled water was used for conditioning the samples at 60 ± 1 °C. Samples were conditioned for a period of two months, ensuring full saturation with water (3.44 wt %) [6,24]. Samples were taken out of the water bath and analyzed.

### 3.3. Glass Transition Measurements

Glass transition measurements were conducted using Netzsch GABO Eplexor equipped with a 1.5 kN load cell (Netzsch GABO Instruments, Ahlden, Germany). The glass transition temperature *T*_g_ was determined from the DMTA according to ISO 6721 [21] as the crossing of the tangents of the lower inflection points in the storage modulus curves. *T*_g_ of both dry and saturated (plasticized or wet) samples was determined at three measurement frequencies (0.1, 1, and 10 Hz), to check the influence of testing frequency on the results.

### 3.4. Creep Measurements

Creep tests for the dry and saturated epoxy polymer samples were carried out using a creep testing machine at a constant stress level (10 MPa). Short-term creep tests of 3 h were conducted under various constant temperatures, as in [13]. Creep measurements were conducted using Netzsch GABO Eplexor equipped with a 1.5 kN load cell (Netzsch GABO Instruments, Ahlden, Germany).

### 3.5. TTSP and TTPSP

A MATLAB (MathWorks, Natick, MA, USA) code was developed to generate smooth master curves numerically in accordance with Honerkamp [25]. In the routine, the following function was minimized with respect to the horizontal shifting parameter *a*(*T*) for each shifted time segment

(3) $$err=\frac{1}{n}\overset{n}{\underset{i=1}{\sum }}\frac{{\left(y_{i}\left(t_{i}\right)-\hat{y_{i}}\left(a\left(T\right),t_{i}\right)\right)}^{2}}{{\left(y_{i}\left(t_{i}\right)\right)}^{2}}$$

Here *y_i_* are the experimental values of compliance *D* at times *t_i_*, $\hat{y_{i}}$ are the values of the creep compliance function interpolated by a polynomial and *n* is the number of measurement points in each segment. No vertical shifting was found to be necessary. Since the loading time in the machine used is finite and affects the measurements at short times, the data corresponding to the first 10 seconds in each segment were ignored. The advantage of determining the shift factors with this equation is that the shift is determined purely experimentally. The experimental shift factors can subsequently be compared to viscoelastic theories.

## 4. Results and Discussion

### 4.1. Compliance Master Curves of Dry and Saturated Epoxy Time–Temperature Superposition Principle

**Figure 3:**
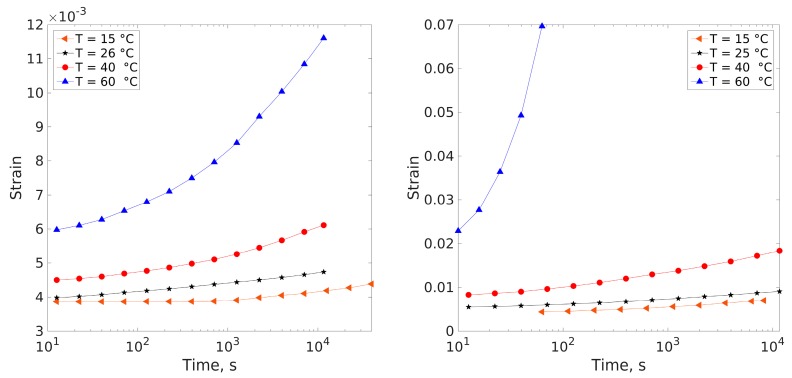
Variation of engineering strain with time (creep) for dry (**left**) and wet (**right**) epoxies subjected to a constant tensile stress of 10 MPa.

The creep curves for dry and for saturated epoxy samples obtained in the experiments are depicted in Figure 3.

The strain vs. time curves were converted into creep compliance vs. time curves simply by dividing the strain by the constant applied stress. Using the well-established time–temperature superposition principle (TTSP), it is now possible to obtain two creep compliance master curves— one for the dry material, and one for the plasticized (saturated with water) material.

**Figure 4:**
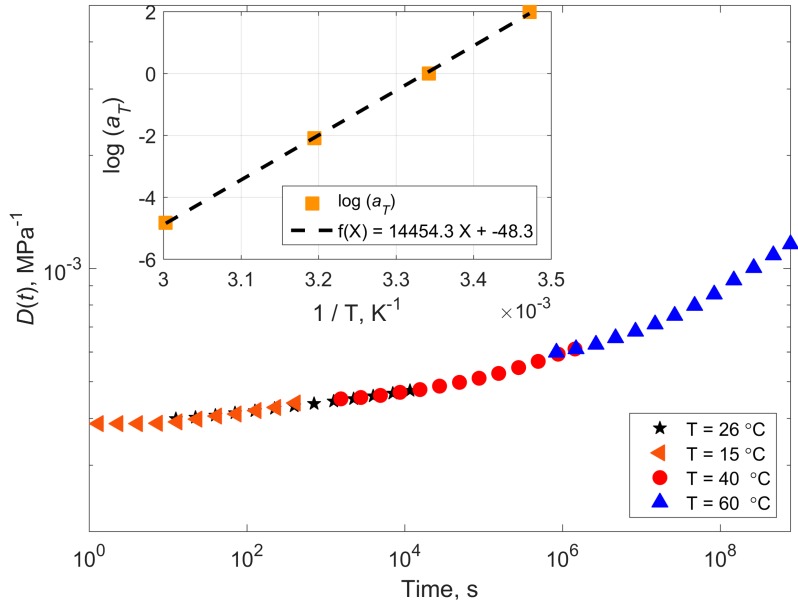
Creep compliance master curve of the dry epoxy. *σ* = 10 MPa, *T*_ref_ = 26 °C. The inset shows variation of the shifting factor with inverse temperature and its linear fit with the corresponding equation. The activation energy calculated using Arrhenius equation is 277 kJ/mol.

The creep compliance master curve of the dry epoxy at 10 MPa applied stress is shown in Figure 4 (*T*_ref_ = 26 °C). The ultimate tensile strength of this epoxy is 60.5 ± 2.7 MPa, after [6]. The experimentally determined best shift factors according to Equation (3) are listed in Table 1. The corresponding activation energies according to Equation (1) are also listed in Table 1.

**Figure 5:**
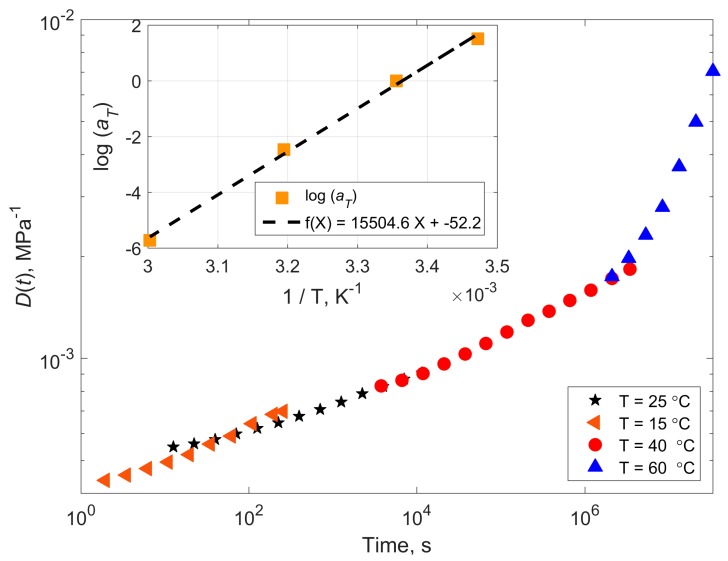
Creep compliance master curve of the saturated epoxy. *σ* = 10 MPa, *T*_ref_ = 25 °C. The inset shows variation of the shifting factor with inverse temperature and its linear fit with the corresponding equation. The activation energy calculated using Arrhenius equation is 297 kJ/mol.

The creep compliance master curve of the saturated (wet) epoxy at 10 MPa applied stress is shown in Figure 5 (*T*_ref_ = 25 °C). Ultimate tensile strength of wet epoxy is 48.5 ± 3.3 MPa, when saturated with water, after [6]. The experimentally determined best shift factors according to Equation (3) are also listed in Table 1, together with the corresponding activation energies.

It is evident that in both cases (dry and wet) TTSP applies reasonably well, and the individual creep segments sufficiently overlap enabling smooth master curves. As expected, temperature has a profound effect on the material modulus and compliance, leading to a relatively large compliance magnitude and, thus, excessive deformation at long times and the highest temperature in the experiment. The water saturated epoxy exhibits greater compliance values than the virgin material due to plasticization. Furthermore, the wet epoxy experienced stress rupture at 60 °C which was not observed in the dry material at the same temperature and stress level. The wet data show higher scatter of the shift factors than the dry data. This is most likely due to nonlinear effects, being very clear at the high temperature tests that failed due to rupture during testing.

The behavior of the shifting factors with inverse temperature (1/*T*) can be described by a linear relationship with a good accuracy in both cases. This confirms applicability of Arrhenius type of equations to the problem.

The calculated activation energy for the dry material is 277 kJ/mol, while the one for the wet material is 297 kJ/mol. The two values are the same within the experimental scatter. When looking at the list of activation energies in Table 1 more closely the activation energies increase for higher temperatures and towards higher strains. The increase is an indication that other deformation mechanisms become important. At strains of more than 1%, nonlinear viscoelasticity or even plastic deformations may become important. They would not be covered by the simple Arrhenius equation given in Equation (1).

### 4.2. Dry-Plasticized Compliance Master Curve Time–Temperature–Plasticization Superposition Principle

**Figure 6:**
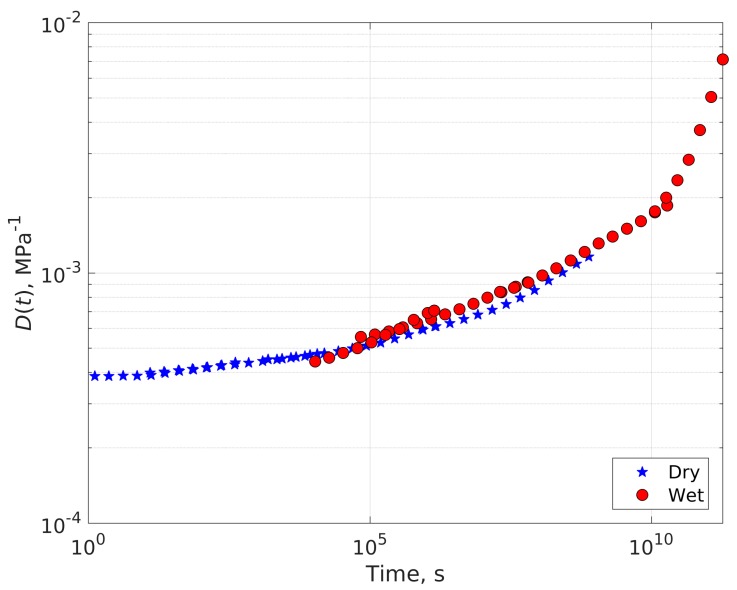
Complete dry-to-wet creep compliance master curve. Dry material as a reference state. *σ* = 10 MPa.

The creep compliance master curves of dry and saturated epoxy at the chosen stress level of 10 MPa will be used for the further analysis. Figure 6 shows that the two curves can be reasonably well superimposed on each other by horizontal shifting. Since the two activation energies for the dry and wet shift factors can be seen as rather similar, the same viscoelastic processes seem to apply for dry and saturated epoxy. The limitations will be discussed below. The shift factor was found experimentally to be $\log a_{dry-to-plast}$ = −3.74, using the optimization from Equation (3).

### 4.3. Predicting the Creep Characteristics of the Plastizised Epoxy from the Dry Data

Following Equation (2) it should also be possible to analytically predict the shift factor based on the different glass transition temperatures for the dry and wet material.

**Figure 7:**
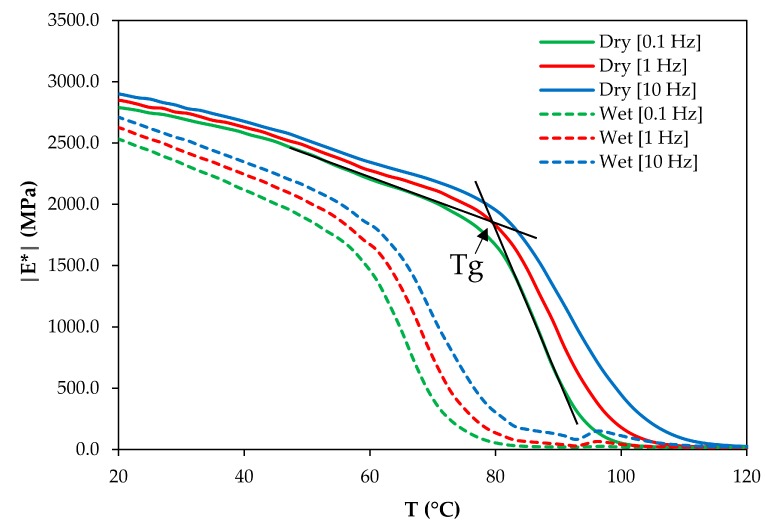
Complex storage moduli of dry and saturated epoxy samples at various testing frequencies (0.1, 1, and 10 Hz). An example of *T*_g_ determination is indicated with black lines and an arrow.

The glass transition temperature *T*_g_ of dry and saturated epoxy was measured by DMTA analysis at a test frequency of 0.1 Hz, 1 Hz, and 10 Hz. The DMTA data are shown in Figure 7.

In materials science *T*_g_ is often defined as the midpoint of the transition zone-defined by the turning point of the slope of the curve. The *T*_g_ values were obtained according to the standard ISO 6721 [21]. They are listed in Table 2. The shift factors calculated according to TTPSP (Equation (2)) are also listed in Table 2.

The material’s *T*_g_ becomes larger at higher test frequencies, as is the case for most polymer systems. The *T*_g_ determination method involves some measurement accuracy, which is included in the Table 2. Standard deviation of the predicted dry-to-plasticized shift factor involves both the *T*_g_ measurement uncertainty (±2 °C) and the standard deviation of activation energy (±16 kJ/mol), which was reported in Table 1.

Using the activation energy of the dry material 277 kJ/mol and the dry and wet *T*_g_ values in Equation (2) the shift factor log ***a_dry-to-plast_*** can be calculated. The factors are listed in Table 2. Even though the difference between glass transition temperatures of dry and wet materials is fairly independent of testing frequency, the shift factors increase for lower test frequencies due to the nonlinear nature of Equation (2).

The lowest testing frequency used in the DMTA experiments was 0.1 Hz. Creep is a long-time (or very low frequency) process. Ideally, *T*_g_ values measured at frequencies of the order of 10^−3^ to 10^−4^ Hz or even lower should be used for calculating the shift factor. Since the equipment cannot test at such low frequencies, the measurement at the lowest test frequency is taken here for comparing the predicted and experimental shift factor. For the lowest test frequency of 0.1 Hz, the glass transition temperatures are *T*_g_ dry = 82 ± 2 °C and *T*_g_ wet = 57 ± 2 °C. In this case, the predicted shift factor is −3.09 ± 0.70. The predicted factor is somewhat lower than the experimental value of −3.74. The experimental factor, however, falls within the somewhat high scatter of the predicted log *a**_dry-to-plast_*** values (standard deviations are shown in Table 2).

**Figure 8:**
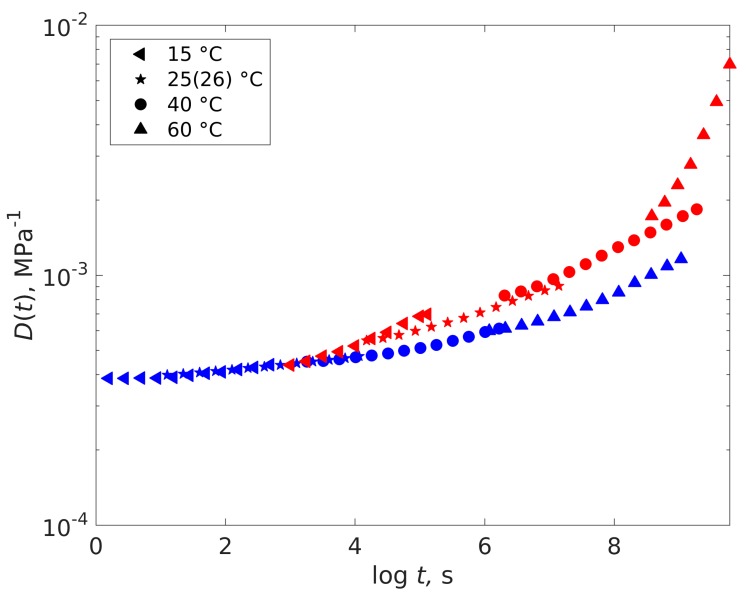
Complete dry compliance master curve and superimposed wet master curve calculated by using the activation energy of the dry material and the *T*_g_ of the dry and wet material. *σ* = 10 MPa. Blue and red curves represent dry and wet compliance master curves, respectively.

Using the activation energy of the dry epoxy of 277 kJ/mol for making a TTSP master curve of the wet material and using the shift factor from dry to wet based on the *T*_g_ difference, ideally the combined dry wet master curve should be obtained. The result is shown in Figure 8.

The dry and master curves do not superimpose as well as using the experimental shift factors, see Figure 6. The wet data fall reasonably well on to the dry master curve for low temperatures. However, they clearly deviate for high temperatures. There are several reasons for the discrepancy. The *T*_g_ values should be obtained at lower frequencies, this would increase the shift factor, giving a better fit. Furthermore, the wet material reaches strains of more than 1% where typically nonlinear effects and plasticity become important. Nevertheless, it can be seen that the *T*_g_ shift due to plasticization describes the shift of the master curves in principle and the *T*_g_ shift should be responsible for the change in properties.

For good quantitative agreement, the experimental shift factor obtained from the creep curves should be used and then the wet and dry creep curves can be described by a single master curve as shown in Figure 6.

The master curve is only valid below *T*_g_ for the epoxy investigated here. The rupture of the wet epoxy during the creep test close to the glass transition temperature demonstrated that nonlinear effects become important that are not properly addressed anymore by the method. It is likely that the method described here works for all polymers below the glass transition temperature. It is important to note that *T*_g_ drops significantly for the epoxy saturated with water, affecting also the validity of the master curve.

## 5. Conclusions

A time–temperature–plasticization superposition TTPSP master curve was obtained for the creep compliance of a dry and plasticized (saturated with water) amine-based epoxy.

The dry epoxy exhibited thermo-rheological simple behavior and the creep curves obtained at 15, 26, 40, and 60 °C could be nicely shifted into a master curve using the Arrhenius approach. The epoxy saturated with water had a higher compliance as expected for a plasticized material. A master curve of the plasticized material’s compliance could be obtained using the same approach as for the dry material. The activation energies in the Arrhenius equation were the same within the experimental error for both cases. Subsequently, the two master curves could be combined to a dry-plasticized master curve also giving good overlap of the data.

The shift factor from the dry to the plasticized curve is also roughly given by the Arrhenius equation using the activation energy of the dry (or wet) material and the difference of the glass transition temperatures for the dry and plasticized material. The discrepancy between the experimental and theoretical shift factor is seen to be due to obtaining the *T*_g_ at higher testing rates than the very slow creep rate. Some nonlinear effects may also influence the results at high temperatures and high strains.

This methodology enables prediction of long-term viscoelastic behavior of plasticized amorphous polymers at temperatures below the glass transition temperatures *T*_g_ based on the short-term creep experimental data of the respective dry material and the difference between *T*_g_ values of dry and plasticized polymer. Furthermore, the *T*_g_ of the plasticized epoxy can be predicted with reasonable accuracy using the *T*_g_ of the dry material (*T*_g_ dry) and the shift factor due to plasticization (log ***a_dry-to-plast_***).

The dry–wet (dry–plasticized) master curve enables prediction of long-term creep of the epoxy polymer. This work provides an accelerated testing methodology for predicting the time-dependent mechanical properties of plasticized amorphous polymers below the *T*_g_. The method depends on the glass transition *T*_g_ measurement frequency. The method is most precise at the lowest *T*_g_ determination test frequencies.

## Figures and Tables

**Table 1:** Experimentally determined TTSP shift factors for creep compliance of dry and wet epoxy. The mean is obtained as the best fit of the curves in Figure 4 and Figure 5. StDev stands for standard deviation.

Temperature (°C)	Shift Factor Log *a*(*T*)		Activation Energy (kJ/mol)	
	Dry Epoxy	Wet Epoxy	Dry Epoxy	Wet Epoxy
15	1.98	1.51	297	227
26 (*T*_ref_)	0	0	-	-
40	−2.09	−2.47	268	316
60	−4.82	−5.72	271	321
		Mean	277	297
		StDev	16	53

**Table 2:** Experimentally determined *T*_g_ values and best TTPSP shift factors

*T*_g_ Determination Frequency (Hz)	Glass Transition Temperature *T*_g_ (°C)		Dry-to-Plasticized Shift Factor log *a_dry-to-plast_*	
	Dry Epoxy	Wet Epoxy	Predicted Using TTPSP, According to Equation (2)	Obtained Experimentally, According to Equation (3)
0.1	82 ± 2	57 ± 2	−3.09 ± 0.70	−3.74
1	83 ± 2	60 ± 2	−2.81 ± 0.68	
10	84 ± 2	62 ± 2	−2.66 ± 0.67	
